# Structured multi-domain EEG descriptors with phase-based connectivity for lie and truth detection

**DOI:** 10.1016/j.mex.2026.104077

**Published:** 2026-07-28

**Authors:** Dwi Utari Surya, Sholeh Hadi Pramono, Panca Mudjirahardjo, Muhammad Aziz Muslim, Cries Avian, Mahdin Rohmatillah

**Affiliations:** a Department of Electrical Engineering, Faculty of Engineering, Universitas Brawijaya, Malang, 65145, Indonesia; b Department of Creative and Digital Industry, Faculty of Vocational Studies, Universitas Brawijaya, 65145, Indonesia

**Keywords:** EEG-based Lie and Truth detection, Multi-domain descriptors, Phase connectivity, Handcrafted EEG features

## Abstract

Wearable EEG-based Lie and Truth detection requires structured descriptor engineering to achieve reproducible results and ensure method transparency. The existing approaches use loosely integrated features alongside hidden modeling methods, which create challenges for understanding their results and verifying research progress across different studies. The existing research gap requires the development of a deterministic descriptor framework to document wearable EEG systems.

The research presents the Structured Multi-Domain EEG Descriptor with Phase-Based Connectivity as a reproducible framework that uses five-channel wearable EEG signals to classify binary Lie-Truth. The method emphasizes coordinated descriptor organization and controlled evaluation, summarized as follows:

• The structured integration of temporal statistics, fractal complexity, spectral power distributions, and phase-based connectivity within a unified descriptor representation augmented by meta-correlation modelling.
• The processing framework provides detailed specifications for preprocessing, window-level segmentation, descriptor extraction, fold-wise normalization, classifier evaluation, and computational profiling.
• A controlled evaluation strategy for examining statistical consistency, descriptor separability, domain-level feature contribution, and workstation-based computational feasibility.

Validation on the publicly available LieWaves dataset demonstrates stable fold-wise behaviour and consistent classifier responses under controlled conditions.

## Graphical abstract

**Figure:**
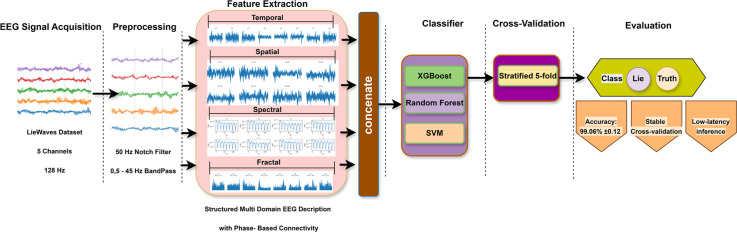

**Specifications table**

**Table:** 

**Subject area**	Neuroscience
**More specific subject area**	*Biomedical signal processing; EEG analytics; Lie and Truth detection using physiological signals; machine learning*
**Name of your method**	***Structured Multi-Domain EEG Descriptors with Phase-Based Connectivity***
**Name and reference of original method**	*C. Avian et al., “Temporal Spatial and Signal Descriptor Feature Extraction for Electronic Nose Signal Processing,” IEEE Sens. J., 2024.*
**Resource availability**	*Aslan, M., Baykara, M., & Alakus, T. B. (2024). LieWaves: Dataset for lie detection based on EEG signals and wavelets. Medical & Biological Engineering & Computing.* *Repository: Mendeley Data.* DOI: *10.17632/5gzxb2bzs2.2* All experiments were conducted on a workstation equipped with an Intel® Core™ i5-14400F processor and 32 GB of RAM, running Windows 11. The system included an NVIDIA GeForce RTX 4090 GPU with 24 GB of VRAM. The motherboard configuration used B760M K V2 DDR4. The development environment was built using Python 3.9 with the Anaconda distribution and the Spyder IDE. This study used the NumPy, SciPy, and MNE libraries to preprocess signals and extract features, and also machine learning models through scikit-learn and XGBoost libraries. To support reproducibility, the implementation scripts and configuration files are publicly available at https://github.com/dutarisurya/lie-truth-eeg-descriptor.

## Background

Electroencephalography (EEG) is widely used for cognitive state decoding due to its high temporal resolution, non-invasiveness, and compatibility with wearable acquisition systems. In Lie and Truth condition classification, EEG provides temporally sensitive physiological measurements that may capture task-related signal variations associated with cognitive load, attention, response preparation, and other experimental processes under controlled conditions [1]. Publicly available wearable datasets, such as LieWaves, have enabled the systematic development of EEG-based lie-detection pipelines, with reported classification accuracies frequently exceeding 90% under controlled paradigms [2,3].

Despite these advances, several methodological limitations persist. First, many deception detection frameworks rely on single-domain feature representations, such as event-related potentials (ERPs), spectral band power distributions, or connectivity measures, which are analyzed independently [[4], [5], [6]]. ERP-based approaches capture stimulus-locked neural responses but are often sensitive to inter-subject variability. Spectral features quantify oscillatory redistribution across frequency bands but may fail to capture nonlinear dynamics present in EEG signals. Connectivity metrics describe synchronization patterns but are often applied without structured integration with temporal or nonlinear descriptors.

To address these limitations, some studies employ multi-domain feature extraction. However, descriptors from different domains are commonly combined via naive feature concatenation, in which independently extracted features are simply merged into a single vector. While this strategy increases feature diversity, it does not explicitly represent relationships between domains. Consequently, interactions among temporal dynamics, spectral distributions, and spatial synchronization patterns are implicitly modeled by downstream classifiers rather than encoded in the feature representation itself [7].

Second, deep learning models, including convolutional and graph convolutional networks, have demonstrated competitive performance in EEG-based Lie and Truth detection [[8], [9], [10], [11]]. However, these models typically require large training datasets, incur higher computational cost, and often provide limited interpretability. In wearable and forensic contexts, methodological transparency, reproducibility, and computational efficiency are critical operational requirements [12,13].

Third, recent benchmarking analyses highlight reproducibility concerns in Lie and Truth detection pipelines. Therefore, the present results are interpreted within the scope of the applied window-level evaluation protocol [2]. In addition, segmentation strategies such as overlapping sliding windows are often treated as simple preprocessing steps, despite their substantial influence on representational density and class separability [14].

Motivated by these challenges, this work introduces a structured multi-domain EEG descriptor engineering framework. Instead of relying on naive feature concatenation, the proposed method organizes handcrafted descriptors into coordinated feature domains and incorporates explicit interaction modeling between selected descriptors. The framework integrates four complementary descriptor groups: (i) temporal statistics and Hjorth parameters; (ii) spectral band power and entropy estimated using Welch power spectral density; (iii) fractal complexity measures capturing nonlinear dynamics; and (iv) phase-based connectivity descriptors derived from Hilbert-transform-based weighted Phase Lag Index.

To capture cross-domain dependencies, a log-transformed meta-correlation descriptor is introduced. This mechanism computes the correlation between the structured temporal–spatial descriptor vector and its logarithmically scaled representation, enabling the modeling of interactions between descriptor magnitudes and their nonlinear scaling behavior without substantially increasing feature dimensionality.

The objective of this article is to provide a fully reproducible methodological pipeline for structured EEG descriptor engineering, including segmentation configuration, descriptor formulation, cross-validation control, statistical validation, and computational profiling.

## Method details

**Fig. 1:**
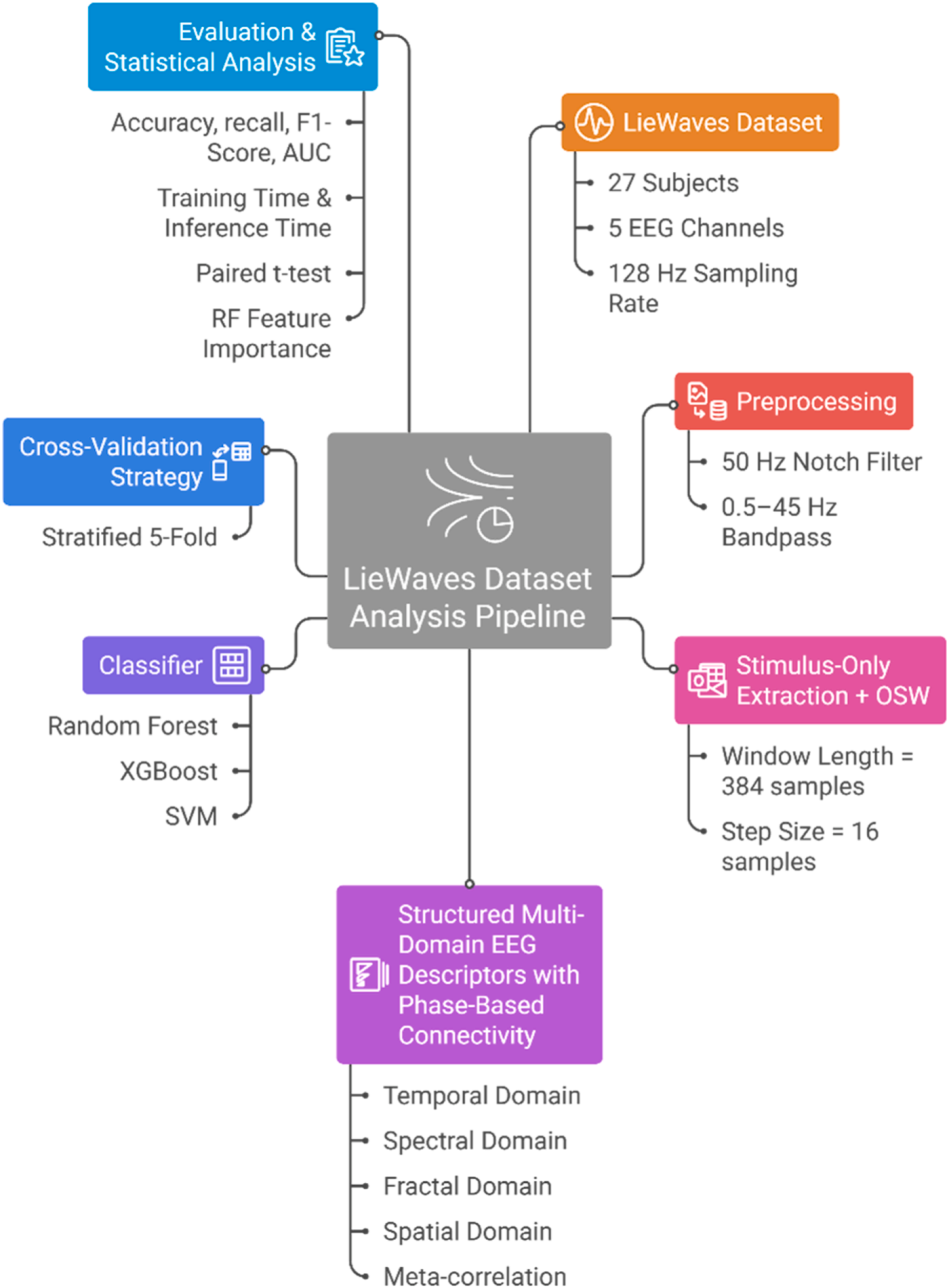
A Deterministic Pipeline Approach to Engineering Structure Multi-domains EEG Descriptors Combined with Phase-Based Connectivity.

The article presents a complete deterministic system that enables the replication of their binary Lie and Truth decoding work using five-channel EEG data from the LieWaves dataset. The methodology emphasizes (i) controlled signal preprocessing, (ii) structured multi-domain handcrafted descriptor engineering, (iii) stratified cross-validation with training-fold-based normalization, and (iv) statistical and interpretability validation. The study defines all implementation parameters to enable reproducing the results. The complete outputs include confusion matrices, fold-wise paired t-test statistics, LDA separability visualization, feature-importance reports, and ablation summaries as captured in Figure 1.

To improve reproducibility, the proposed pipeline is described as a sequence of deterministic processing stages: EEG loading, 50 Hz notch filtering, 0.5–45 Hz Butterworth bandpass filtering, OSW segmentation, overlapping sliding-window segmentation, multi-domain descriptor extraction, meta-correlation construction, feature concatenation, fold-wise z-score normalization, classifier training, and performance evaluation. All feature-scaling parameters are estimated only from the training folds and applied to the corresponding held-out folds.

### Dataset

The study used EEG data from the publicly accessible LieWaves dataset, which provides multichannel wearable recordings for Lie and Truth detection research [10]. LieWaves was selected as the primary dataset because it is a publicly available EEG dataset specifically designed for Lie and Truth detection, using five-channel wearable EEG recordings (AF3, T7, Pz, T8, AF4) at a sampling frequency of 128 Hz, and contains recordings from 27 participants. The five-channel configuration follows the original LieWaves wearable EEG acquisition protocol. Therefore, the present study does not use a high-density EEG montage but instead evaluates the proposed descriptor framework within the low-channel wearable EEG setting defined by the dataset.

The recording session lasts 75 seconds and contains 25 trial blocks, which repeat. Each trial has a total duration of 3 seconds, starting with a 1-second baseline period on a black screen, followed by 2 seconds of stimulus presentation. The system uses a binary system to provide session-level labels, which include Lie as 0 and Truth as 1.

The fixed electrode configuration ensures consistent spatial ordering across subjects, enabling the calculation of connection measurements between brain areas.

All segmentation, preprocessing, and feature extraction procedures described in the following sections are applied uniformly to all subjects as illustrated in Table 1.

**Table 1:** Liewaves Dataset and Experimental Configuration.

**Property**	**Description**
Number of Subjects	27
EEG Channels	5 (AF3, T7, Pz, T8, AF4)
Sampling Frequency	128 Hz
Recording Duration	75 seconds per session
Trials per Session	25 trials
Trial Structure	3 seconds per trial
EEG Segment Configuration	Overlapping Sliding Window (OSW): 384 samples (3 s) with 16-sample step
Total Window Samples	31158 window segments
Class Labeling	2 classes: Truth (1) and Lie (0)
Evaluation Strategy	Stratified 5-fold Cross-Validation
Classifiers Evaluated	Random Forest, XGBoost, Support Vector Machine
Performance Metrics	Accuracy, Recall, F1-score, AUC, Training and Inference Time, Paired t-test, LDA, RF Feature Importance

### Signal preprocessing

Raw EEG recordings are processed using a deterministic two-stage filtering procedure applied independently to each channel. First, a 50 Hz notch filter is applied to suppress power-line interference [15]. The notch filter is implemented with a quality factor to ensure narrowband attenuation centered at 50 Hz without affecting adjacent frequency components. Second, a fourth-order Butterworth bandpass filter with cut-off frequencies of 0.5 Hz and 45 Hz is applied [14]. The lower bound (0.5 Hz) removes slow baseline drift, while the upper bound (45 Hz) attenuates high-frequency noise and muscle artifacts. However, it does not include re-referencing, ICA-based artifact correction, EOG/EMG-informed correction, or manual artifact rejection.

This choice was made to maintain a fully deterministic and reproducible descriptor-extraction pipeline without introducing subjective component selection or dataset-specific rejection thresholds. Nevertheless, this preprocessing choice is not artifact-neutral, particularly in frontal and temporal electrodes. The study used the LieWaves dataset, which was collected during controlled experiments that produced continuous EEG data from wearable devices. This study implements a basic preprocessing method that uses notch and bandpass filters to eliminate primary noise sources while maintaining original signal attributes.

### Session-level EEG segmentation

Each recording session consists of 25 consecutive trials. Each trial has a duration of 3 seconds (384 samples at 128 Hz). Therefore, each trial corresponds to 384 samples, and the complete 75-second session contains 9600 samples per channel. After deterministic filtering, the full session-level EEG signal is retained and segmented directly using an overlapping sliding window.

The filtered session-level EEG signal is represented as:

(1) $$X\in R^{T\times C}$$

where X represents the filtered EEG signal, T=9600 denotes the number of time samples in a 75-second recording, and C=5 denotes the number of EEG channels, corresponding to AF3, T7, Pz, T8, and AF4.

All stimulus segments are concatenated sequentially. The matrix is then transposed to channel-major format

(2) $$X_{ch}\in R^{C\times T}.$$

### Overlapping sliding window (OSW) segmentation

To generate fixed-length learning instances while preserving temporal continuity, an overlapping sliding window strategy is applied to the session-level EEG signal [16,17].

Window parameters are defined as:

• Window length (L)=384 samples
• Step size (S)=16 samples

For each shift k, a window is defined as:

(3) $$X_{win}^{\left(k\right)}\in R^{5\;\times \;384}$$

(4) $$X_{win}^{\left(k\right)}=X_{ch}\left[:,\;kS:\;kS+L\right]$$

where $X_{win}^{\left(k\right)}$ denotes the k-th segmented EEG window, $X_{ch}\in R^{C\times T}$ denotes the channel-major session-level EEG matrix, C = 5 is the number of EEG channels, T = 9600 is the number of time samples in a 75-second recording, L = 384 is the window length, S = 16 is the step size, and k denotes the window index.

The segmentation procedure is applied uniformly across all sessions and subjects without adaptive tuning. The window length is 384 samples, corresponding to 3 seconds at 128 Hz, and the step size is 16 samples, corresponding to 0.125 seconds. This configuration produces 95.83% overlap between adjacent windows. This configuration is intended to generate dense window-level EEG representations for controlled analysis of descriptor separability. Using this configuration, the complete dataset consists of N=31,158samples. Each window is processed independently during feature extraction.

### Feature extraction framework: structured multi-domain eeg descriptors with phase-based connectivity

**Fig. 2:**
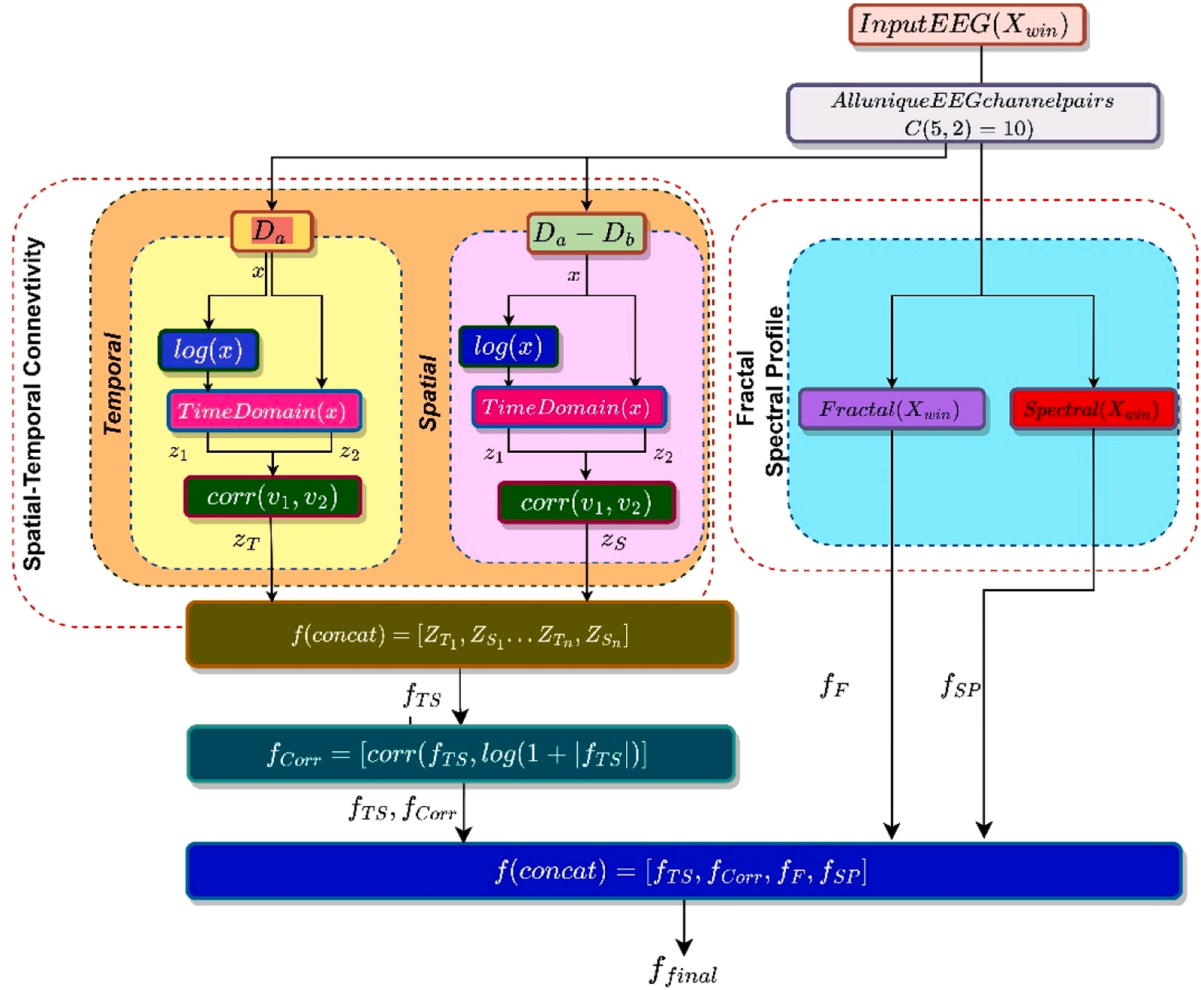
Structured Multi-Domain Descriptor Construction with Phase-Based Connectivity and Meta-Correlation Integration.

The structured multi-domain handcrafted descriptor is constructed for each segmented EEG window as shown in Fig. 2. The objective of this design is to organize feature extraction into coordinated domains rather than relying on unstructured concatenation. For each EEG window

(5) $$X_{win}\in R^{C\times L}$$

where C=5channels and L=384samples.

For a deterministic 106-dimensional feature vector is constructed as

(6) $$f_{final}=\left[f_{T},f_{S},f_{F,\;}f_{SP\;}\right]$$

Where $f_{TS}$ denotes temporal-statistical descriptors, $f_{F\;}$denotes fractal descriptors, $f_{SP\;}$denotes spectral descriptors and $f_{corr}$ denotes spatial connectivity descriptors. The mathematical formulations and variable definitions for each feature domain are summarized in Table 2.

**Table 2:** Mathematical Formulations and Variable Definitions for Each Domain-Specific Feature Set Included in The Structured Multi-Domain EEG Descriptors with Phase-Based Connectivity Framework.

Domain	Formula	Variable Meaning
Temporal	$\mu_{a}=\frac{1}{N}\sum x_{a,i},\sigma_{a}=\sqrt{\frac{1}{N-1}\sum {\left(x_{a,i}-\mu_{a}\right)}^{2}\;}$ $skew_{a}=\frac{1}{N}\sum_{i=1}^{N}{\left(\frac{x_{a,i}-\mu_{a}}{\sigma_{a}}\right)}^{3}$, $kurt_{a}=\frac{1}{N}\sum_{i=1}^{N}{\left(\frac{x_{a,i}-\mu_{a}}{\sigma_{a}}\right)}^{4}$	$x_{a}$: EEG signal of channel a, N: Number of samples, $\mu_{a}$: Mean amplitude, $\sigma_{a}$: Standard deviation, $skew_{a}$: skewness, $kurt_{a}$:kurtosis
	$A_{a}=Var\left(x_{a}\right),\;M_{a}=\;\sqrt{\frac{Var(x{{}^{\prime }}_{a})}{Var(x)}},\;C_{a}=\;\frac{M^{\prime }}{M}$	$A_{a}$: Hjorth Activity, $M_{a}$: Mobility, $C_{a}$: Complexity
Spectral	$BP_{abs}=\sum_{f\in bands}PSD_{a}\left(f\right)$ $BP_{rel}=BP_{abs}/\sum BP_{abs}$ $H_{a}=-\sum_{f}P\left(f\right)logP\left(f\right),\;P\left(f\right)=\frac{PSD_{a}\left(f\right)}{\sum PSD_{a}\left(f\right)}$	$BP_{rel}$: Relative bandpower, $BP_{abs}$: Absolute bandpower, $H_{a}$: Spectral entropy, $PSD_{a}\left(f\right)$: Power spectral density
Spatial	$wPLI_{a,b}=\;\frac{|E[Im(Z_{a,b})]|}{E[|Im(Z_{a,b})|]}$ $\Delta_{ab}=\;\mu_{a}-\mu_{b}$	$wPLI_{a,b}$: weighted Phase Lag Index between channels, $\Delta_{ab}$: Mean amplitude difference, $\mu_{a}$, $\mu_{b}$: mean amplitude of channels a,b
Fractal	$D_{a}=\frac{\text{log}(N)}{\log\left(N\right)+\text{log}\left(L_{a}/N\right)},\;L_{a}=\sum_{i}\left|x_{a,i+1}-x_{a,i}\right|$	$D_{a}$: Fractal dimension, $L_{a}$: Curve length, $x_{a,i}$: i-th sample of channel a
Corr	$cov\left(x_{a},x_{b}\right)=\frac{1}{N-1\;}\sum_{i=1}^{N}\left(x_{a,i}-\bar{x_{a}}\right)\left(x_{b,i}-\bar{x_{b}}\right)$ $Corr_{a,b}=\frac{Cov(x_{a},x_{b})}{\sigma_{a}\sigma_{b}}$	$Cov(x_{a},x_{b})$: sample covariance between signals $x_{a}\;\text{and}\;x_{b}$, $\sigma_{a},\sigma_{b}$: standard deviations of signals $x_{a}$and $x_{b}$ and $Corr(x_{a},x_{b}\;)\;$are normalized linear dependency between the two signals.
Meta-Corr	$f_{corr}=Corr(f_{TS},\text{log}\left(1+|f_{TS}|\right)$	$f_{corr}\;$is meta-correlation descriptor capturing nonlinear scaling relationships between descriptor magnitudes. $f_{TS}$: concatenated temporal–spatial descriptor vector $f_{TS}=\left[f_{T}+f_{S}\right].\;$

The full 106-dimensional descriptor is retained to preserve the complete structured representation of temporal, fractal, spectral, connectivity, and meta-correlation domains. This design supports reproducibility and enables domain-level contribution analysis. No dimensionality reduction is applied in the present study, allowing the influence of each descriptor group to be evaluated transparently.

In this study, phase-based connectivity and meta-correlation are used as engineered discriminative descriptors. They are included to enrich the feature representation and support classifier-level separation of Lie and Truth windows. Because the dataset contains only five EEG channels, phase-based connectivity is interpreted within the limited spatial coverage of the wearable montage. The connectivity descriptors are therefore used to enrich the classifier-level representation rather than to infer complex brain-network organization.

The extracted features are later classified using a battery of classical ML algorithms, including XGBoost, SVM, RF under both binary classification settings.

(A) Temporal descriptors (per channel)

Temporal descriptors are designed to capture intra-channel distributional and dynamical properties within each window [18]. For each channel signal $x_{a}$ first z−normalized to remove amplitude bias and ensure inter-channel comparability:

(7) $${\tilde{x}}_{a}=\frac{x_{a}-\mu_{a}}{\sigma_{a}}$$

Where $\mu_{a}$and $\sigma_{a}$ denote the mean and standard deviation of channel a

This normalization removes amplitude bias and ensures channel comparability. Seven descriptors are computed: Mean, Standard deviation, Skewness, Kurtosis, Hjorth Activity, Hjorth Mobility, and Hjorth Complexity. Since 7 descriptors are computed for each of the 5 channels, the total temporal feature dimension is 7 × 5 = 35 features. These descriptors quantify central tendency, dispersion, distributional asymmetry, peakedness, and second-order signal dynamics.

(B) Fractal descriptor (per channel)

EEG signals exhibit nonlinear and scale-dependent characteristics that are not fully captured by second-order statistics. To quantify structural irregularity, a curve-length fractal dimension is computed per channel [19]. The given fractal dimension reflects signal roughness and local complexity. The total fractal feature dimension is 1 × 5 = 5 features. The fractal descriptor reflects signal irregularity and scaling behavior.

(C) Spectral descriptors via Welch PSD (per channel)

The analysis of oscillatory activity requires the application of Welch's power spectral density (PSD) estimation method. Welch's method provides stable results within brief time intervals [20]. Per channel, the following descriptors are computed: Absolute bandpower (δ (1-4 Hz), θ (4-8 Hz), α (8-12 Hz), β (12-30 Hz)). These features describe energy redistribution and spectral irregularity. The spectral domain contributes 45 features.

(D) Spatial connectivity (per channel-pair)

Inter-channel relationships are modeled using pairwise descriptors [21]. All unique channel pairs are defined as Ω={(i,j)∣1≤i<j≤5} with ∣Ω∣=10*.* To model inter-channel synchronization and asymmetry, spatial descriptors are computed for all unique channel pairs $(\begin{array}{c} 5\\ 2 \end{array})=10\;pairs$. For each unique channel pair, two pairwise descriptors are extracted: weighted Phase Lag Index (wPLI) and amplitude asymmetry. Because five EEG channels produce C(5,2) = 10 unique channel pairs, the spatial-connectivity domain contributes 20 features.

The wPLI-based descriptors are used as engineered pairwise synchronization features between EEG channels. These descriptors are interpreted as discriminative connectivity-related features rather than direct neurophysiological markers. Given the five-channel wearable montage, these descriptors are not intended to represent comprehensive whole-brain connectivity. The wPLI descriptors are computed within fixed-length EEG windows and therefore represent static window-level summaries of pairwise phase synchronization. They are used as engineered descriptors of connectivity for classifier-level discrimination, not as dynamic connectivity models.

(E) Structured Descriptor and Meta-Correlation

Temporal and spatial descriptors are concatenated $f_{TS}=\left[f_{T},f_{S}\right]\in R^{55}$. Rather than directly concatenating all domain features, a meta-correlation term is introduced to model structured nonlinear interaction $f_{Corr}=corr\left(f_{TS},\text{log}\left(1+|f_{TS}|\right)\right)$. where the logarithmic transformation stabilizes the scale of descriptor magnitudes while preserving relative variations across features.

The meta-correlation descriptor is introduced as a compact engineered interaction feature. It is computed by correlating the structured descriptor vector with its log-scaled magnitude representation. The rationale is that EEG descriptors from different domains may exhibit different value ranges and nonlinear scaling behaviour. The log transformation reduces the dominance of large-magnitude descriptors, while the correlation operation summarizes the consistency between the original descriptor structure and its compressed nonlinear representation. Thus, meta-correlation provides a single interaction feature that captures cross-domain magnitude relationships without substantially increasing dimensionality [22].

This mechanism provides a compact interaction descriptor that summarizes higher-order relationships between temporal, spectral, fractal, and connectivity descriptors without introducing a large number of additional features. In the proposed framework, the meta-correlation contributes a single feature while enabling structured interaction modelling across descriptor domains. The final window-level descriptor is constructed as $f_{final}=\left[f_{TS},f_{corr},f_{F},f_{SP}\right]\in R^{106}$. The total number of 106 features generated in the proposed structured Multi-Domain EEG Descriptors with Phase-Based Connectivity framework is summarized in Table 3.

**Table 3:** Feature Dimensionality of the Structured Multi-Domain EEG Descriptors with Phase-Based Connectivity Framework.

**Feature Group**	**Description**	**Dimension**
Temporal Features	Statistical descriptors (mean, std, skewness, kurtosis) and Hjorth parameters (activity, mobility, complexity) extracted per EEG channel (7 features × 5 channels)	35
Fractal Features	Curve-length fractal dimension computed per EEG channel (1 × 5 channels)	5
Absolute Bandpower	Welch PSD bandpower in delta (1–4 Hz), theta (4–8 Hz), alpha (8–12 Hz), and beta (12–30 Hz) bands per channel (4 × 5 channels)	20
Relative Bandpower	Normalized bandpower per channel (4 × 5)	20
Spectral Entropy	Shannon entropy of normalized PSD per channel (1 × 5)	5
Amplitude Asymmetry	Mean amplitude difference between all channel pairs (C(5,2)=10)	10
Phase-based Connectivity	Weighted Phase Lag Index computed between channel pairs using Hilbert transform (C(5,2)=10)	10
Meta-Correlation (Log)	Correlation between the structured descriptor vector and its log-transformed magnitude representation	1
Total Feature Dimension (Full Descriptor)	$f_{final}=\left[f_{TS},f_{Corr},f_{F},f_{SP}\right]$	106

### Algorithmic workflow of structured multi-domain eeg descriptors with phase-based connectivity

Algorithm 1 summarizes the overall procedural workflow of the Structured Multi-Domain EEG Descriptor with Phase-Based Connectivity. The framework begins with deterministic preprocessing of multichannel EEG signals, followed by full session overlapping sliding window segmentation using a window length of L=384samples and step size S=16samples. Each resulting window $X_{win}\in R^{5\;\times \;384}$is processed independently to ensure window-level reproducibility.

**Algorithm 1:** Structured Multi-Domain EEG Descriptor Extraction and Window-Level Classification for LieWaves EEG.

Step Procedure
Input:
Raw EEG sessions $X_{s}$∈ $R^{TxC}$, T=9600 samples, C=5 channels (AF3,T7,Pz,T8,AF4)
Sampling rate $f_{s}$ = 128 Hz
Label map $Y_{map}$: (subject_id, session_id) → y ∈ {0,1} (Lie=0, Truth=1)
OSW params: window length L=384, step S=16
5-fold StratifiedKFold CV
Output:
metrics: ACC, Recall, F1, AUC, Train/Infer time, confusion matrices
Paired t-test p-value matrix
LDA separability plot
RF feature importance: top-10 + importance by feature groups $\left[f_{TS},f_{Corr},f_{F},f_{SP}\right]$
1: F ← ∅; $y_{w}$← ∅
2: Load label map $Y_{map}$
3: for each session file s do
4: Read EEG X ∈ $R^{9600x5}$ with fixed channel order
5: for each channel ch = 1..C do
6: X[:,ch] ← NotchFilter(X[:,ch], $f_{0}$=50Hz,Q=30)
7: X[:,ch] ← BandpassFilter(X[:,ch], 0.5–45Hz,order=4)
8: End for
9:Transpose to channel-major: $X\leftarrow X^{⊤}\in R^{C\times \;9600}$
10:Generate windows $W=\{X_{\text{win}}^{\left(k\right)}\}$using sliding window with length Land step S, where
$X_{\text{win}}^{\left(k\right)}\in R^{C\times L}$
11: For each window $X_{\text{win}}^{\left(k\right)}\in W$do
12: $f_{\text{final}}^{\left(k\right)}\leftarrow \text{Extract}\left(X_{\text{win}}^{\left(k\right)},f_{s}\right)$
13: $F\leftarrow F\cup \{f_{\text{final}}^{\left(k\right)}\}$
14: $y_{w}\leftarrow y_{w}\cup \left\{Y_{\text{map}}\left(subject_{id},session_{id}\right)\right\}$
15: End for
16: End for
17: Convert Fand $y_{w}$to arrays: $F\in R^{N\times D}$, $y_{w}\in {\left\{0,1\right\}}^{N}$
18: Temporal descriptors (per channel)
19: For each channel c=1..C: normalize $x_{c}\leftarrow \left(x_{c}-\mu_{a}\right)/\left(\sigma_{a}+\epsilon \right)$
20: Compute 7 temporal features: $\left\{\mu_{a},\sigma_{a},skew_{a},kurt_{a},HjorthA_{a},HjorthM_{a},HjorthC_{a}\right\}$
21:Concatenate across channels → $f_{T}\in R^{35}$
22: Fractal descriptors (per channel)
23: Compute curve-length fractal dimension for each channel → $f_{F}\in R^{5}$
24:Spectral descriptors (per channel)
25: Using Welch PSD, compute absolute bandpower $BP_{abs}$(δ,θ,α,β)→ 20dims
26: Normalize to relative bandpower $BP_{rel}$ → 20dims
27: Compute spectral entropy $H_{a}$ → 5dims
28: Concatenate → $f_{SP}\in R^{45}$
29: Spatial–temporal (pairwise) descriptors
30: Define pair set $\Omega =\left\{\left(i,j\right)|1\leq i<j\leq C\right\},|\Omega |=\left(\begin{array}{c} C\\ 2 \end{array}\right)=10$
31: For each (i,j)∈Ω:
32: $\Delta_{ab}\leftarrow \text{mean}\left(x_{a}\right)-\text{mean}\left(x_{b}\right)$*(amplitude asymmetry)*
33: $wPLI_{ab}\leftarrow \text{wPLI}\left(x_{a},x\right)$using Hilbert transform
34: Concatenate all pairs → $f_{S}\in R^{20}$
35: Construct structured descriptor
36: $f_{TS}\leftarrow concatenate\left[f_{T},f_{S}\right]\in R^{55}$
37:Compute meta-correlation descriptor
38: $f_{Corr}\leftarrow corr\left(f_{TS},\text{log}\left(1+|f_{TS}|\right)\right)\in R^{1}$
39: Return final vector
40: $f_{\text{final}}\leftarrow \left[f_{TS},f_{Corr},f_{F},f_{SP}\right]\in R^{106}$
41: Return $f_{\text{final}}$
42: Initialize classifiers: RandomForest (RF), XGBoost (XGB), and SVM
43: Perform StratifiedKFold CV with K=5on $\left(F,y_{w}\right)$
44: For each fold: fit scaler on train only, train models, predict on test fold, compute metrics, store fold metrics
45: Aggregate confusion matrices, paired t-test p-values, RF importances, and ablation results
46: End

For each window, temporal descriptors are first extracted per channel, including statistical measures (mean, standard deviation, skewness, kurtosis) and Hjorth parameters (activity, mobility, complexity), forming the temporal feature vector $f_{T}\in R^{35}$. Fractal dimension features are then computed per channel, yielding $f_{F}\in R^{5}$. Spectral descriptors derived from Welch’s power spectral density estimation generate $f_{SP}\in R^{45}$, comprising absolute bandpower, relative bandpower, and spectral entropy across predefined frequency bands.

To model inter-channel relationships, spatial connectivity measures are computed across all unique channel pairs $\left((\begin{array}{c} 5\\ 2 \end{array})=10\right)$. For each pair, amplitude asymmetry and weighted Phase Lag Index (wPLI) are extracted, resulting in $f_{S}\in R^{20}$. Temporal and spatial descriptors are then concatenated into a structured representation $f_{TS}\in R^{55}$.

A meta-correlation descriptor $f_{Corr}\in R^{1}$is subsequently computed by correlating $f_{TS}$with its log-transformed magnitude $\text{log}(1+|f_{TS}|)$, enabling compact modeling of higher-order nonlinear interactions without dimensional expansion. The final window-level descriptor is constructed as$\;f_{final}=\left[f_{TS},f_{Corr},f_{F},f_{SP}\right]\in R^{106}.$

All extracted descriptors are standardized using parameters estimated exclusively from training folds and subsequently evaluated under stratified 5-fold cross-validation using classical machine learning classifiers. Performance metrics are computed per fold and aggregated after cross-validation.

### Data normalization

Before model training, the extracted 106-dimensional feature matrix is standardized using z-score normalization. The training data of each cross-validation fold is used to estimate normalization parameters, which prevents normalization leakage from the test fold [23]. However, this fold-wise normalization procedure does not eliminate temporal dependencies among highly overlapping windows; therefore, the evaluation is interpreted as window-level descriptor separability in a controlled, subject-dependent setting. The procedure requires that all descriptor domains, including temporal, fractal, spectral, and spatial connectivity and meta-correlation, undergo uniform scaling before the classification process. The transformation process uses training-based parameters to apply the test fold transformation. Each fold undergoes separate standardization, which prevents cross-fold contamination during feature scaling while enabling reproducibility.

### Classification strategy

The structured 106-dimensional window-level descriptor was evaluated using three classical supervised machine learning classifiers: Random Forest, XGBoost, and Support Vector Machine. These classifiers were selected because they represent complementary learning paradigms commonly used for tabular EEG feature classification: Random Forest (bagging-based ensemble learning), XGBoost (regularized gradient boosting), and Support Vector Machine (kernel-based margin classifier). Random Forest produces reliable results through its ensemble bagging, which models complex feature relationships [24], while XGBoost delivers fast boosting-based machine learning through its integrated performance control features [25], and SVM functions as a powerful edge-based automatic system that scientists frequently apply in EEG research [26]. The research assesses the proposed descriptor's ability to distinguish among classes by examining the learning mechanisms used by classifiers. All classifiers were implemented using the Scikit-learn and XGBoost libraries in Python.

The study uses a stratified 5-fold cross-validation method, which maintains the original distribution of Lie (0) and Truth (1) labels throughout the study's different cross-validation folds [27]. This method maintains constant class ratios throughout both training and evaluation. The dataset is divided into training and test sets at each fold. The training dataset serves as the basis for determining feature normalization parameters. The testing subset uses the same normalization parameters established during training, while the model trains only on the training fold and evaluates the model on the held-out fold. The procedure establishes two distinct stages for model development, which separate the training process from the evaluation stage.

Hyperparameter optimization was performed only within the training portion of each cross-validation fold [17]. The system evaluates different setup options during cross-validation to identify the configuration that yields the highest average validation results. The team establishes permanent hyperparameter settings to use across different evaluation rounds. The system requires users to specify random seeds because this process enables controlled execution results. Table 4 presents a summary of the optimal configurations identified as most effective.

**Table 4:** Final Hyperparameter Configuration After Grid Search Configuration.

**Model**	**Parameter Configuration**
XGB	n_estimators = 1200, max_depth = 8, learning_rate = 0.015, subsample = 0.85, colsample_bytree = 0.85, reg_lambda = 1.0, eval_metric = "logloss", random_state = 42
RF	n_estimators = 900, max_features = None, min_samples_leaf = 1, random_state = 42
SVM	kernel = "rbf", C=50, gamma=”auto”, probability = True, random_state = 42

### Evaluation and testing scenario

The evaluation metrics for each fold include accuracy (ACC), recall, F1-score, and area under the ROC Curve (AUC). The metrics show mean values with standard deviations, obtained from multiple test folds [28]. Confusion matrices show how predictions perform across different classes [29]. The statistical method assesses performance at the fold level by using multiple data partitions rather than a single partition. To assess statistical differences between classifiers, paired t-tests are conducted using fold-wise accuracy values. The resulting p-value matrix quantifies pairwise statistical significance across models [30].

The primary validation protocol in this study is window-level stratified 5-fold cross-validation. This protocol evaluates subject-dependent descriptor separability under controlled conditions. A subject-dependent setting evaluates whether the extracted descriptors can discriminate conditions within a calibrated subject or controlled acquisition population.

Feature importance analysis is conducted using the Random Forest model. Importance scores are averaged across folds and reported at two levels: top-k individual features and aggregated domain-level contributions (temporal, spatial, fractal, spectral, meta-correlation) [31]. Additionally, an ablation study is performed by evaluating isolated feature groups independently and comparing them with the complete multi-domain descriptor. This procedure enables structured assessment of domain-wise contributions.

Computational efficiency is evaluated by measuring training time per fold and inference time. Inference latency is averaged across repeated runs to reduce timing variance. All timing measurements are performed under identical computational settings.

## Method validation

### Classification performance

The performance values reported in Table 5 should be interpreted within the scope of the window-level validation protocol on the LieWaves dataset using three classical classifiers: XGBoost (XGB), Random Forest (RF), and Support Vector Machine (SVM). All results are reported as mean ± standard deviation across a stratified 5-fold cross-validation. They quantify the discriminative separability of the proposed descriptors under a controlled subject-dependent evaluation setting. Therefore, the high accuracy values indicate strong window-level separability of Lie and Truth labels in the current dataset configuration, but they should not be interpreted as definitive evidence of subject-independent Lie and Truth classification. The proposed representation contains 106 handcrafted features, and model evaluation is performed using classical machine learning classifiers suitable for structured tabular data.

**Table 5:** Classification Performance and Computational Efficiency.

Model	Acc (%)	Recall (%)	F1 Score (%)	AUC (%)	Training Time (s)	Inference Time (s)
XGB	98.73 ± 0.17	98.56 ± 0.21	98.72 ± 0.17	99.90 ± 0.03	6.4046 ± 0.2109	0.0153s ± 0.0010s
SVM	97.78 ± 0.13	97.73 ± 0.15	97.78 ± 0.13	99.66 ± 0.06	264.4311s ± 20.6280s	4.0603s ± 0.0735s
RF	**99.06 ± 0.12**	98.83 ± 0.23	99.06 ± 0.12	99.96 ± 0.01	7.4777 ± 0.0226	0.1509 ± 0.0084

Random Forest achieved an accuracy of 99.06% ± 0.12, with a recall of 98.83% ± 0.23, F1-score of 99.06% ± 0.12, and AUC of 99.96% ± 0.01. The low standard deviation across folds indicates stable behavior under repeated data partitioning. The near-identical values of accuracy and F1-score suggest balanced classification between Lie and Truth classes. XGBoost achieved 98.73% ± 0.17 accuracy, 98.56% ± 0.21 recall, 98.72% ± 0.17 F1-score, and 99.90% ± 0.03 AUC. The slightly larger variance compared to RF remains within a narrow range, indicating stable window-level performance under the current evaluation setting. Support Vector Machine achieved 97.78% ± 0.13 accuracy, 97.73% ± 0.15 Recall, 97.78% ± 0.13 F1-score, and 99.66% ± 0.06 AUC. Although performance is marginally lower than that of ensemble-based models, the relatively small standard deviation indicates stable convergence across folds under the RBF kernel configuration.

**Fig. 3:**
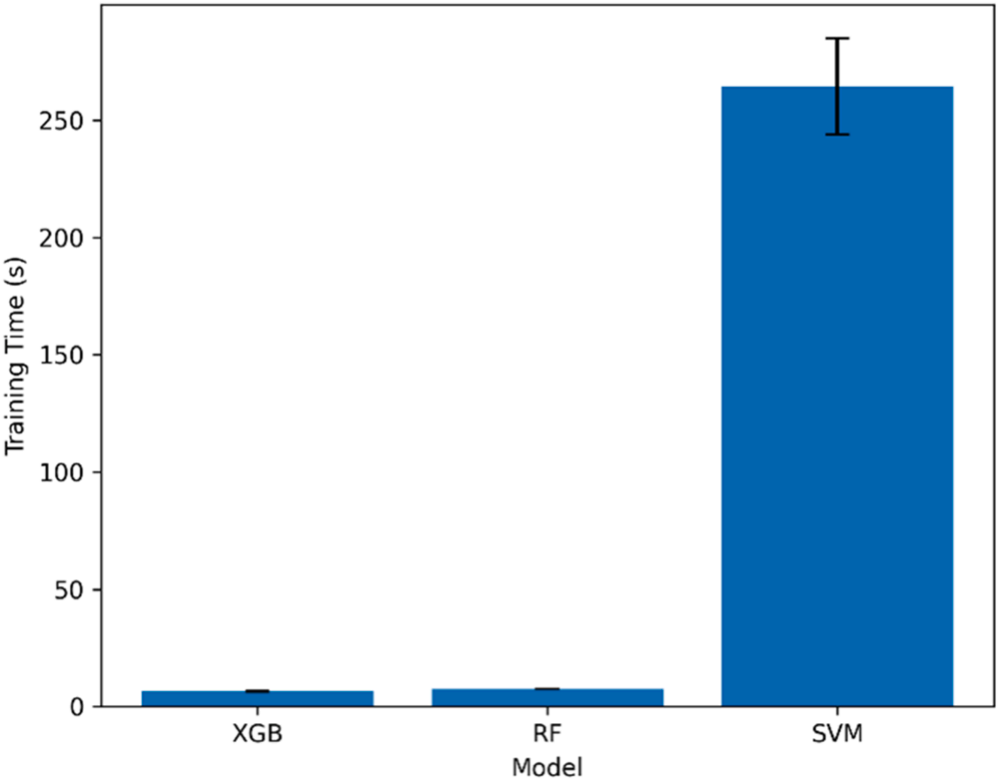
Mean ± Standard Deviation of Training Time Across Classifiers.

The average training time across the different study groups is shown in Fig. 3. The classifiers exhibit significant performance differences. The SVM model takes 264.4311 ± 20.6280 seconds to train because its kernel-based optimization must handle 106 features. The standard deviation indicates that the distribution of data within each fold affects the results at different levels of intensity. The training complexity of ensemble-based methods shows crucial improvements when compared to other training methods. The Random Forest model takes 7.4777 ± 0.0226 seconds to train, while XGBoost completes training 1.0731 seconds faster, at 6.4046 ± 0.2109 seconds. Both models show small standard deviations because their optimization process remained consistent throughout the different cross-validation splits.

**Fig. 4:**
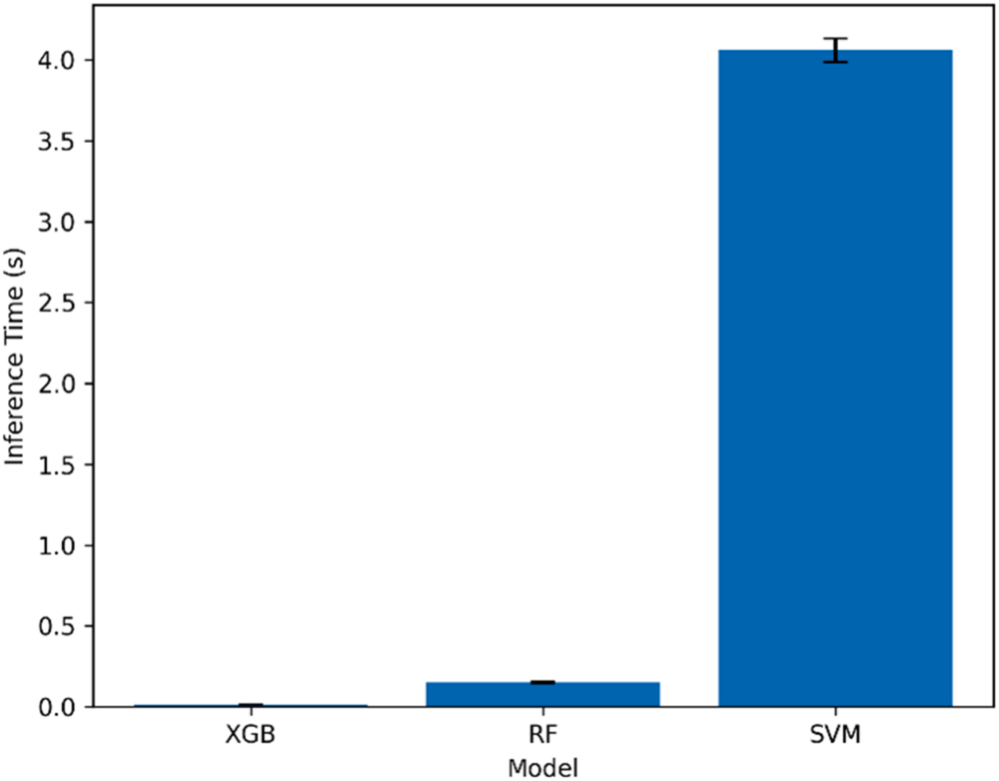
Mean ± Standard Deviation of Inference Time Across Classifiers.

The deployment scenarios show different results because inference latency varies, as shown in Fig. 4. The lowest per-window inference time is achieved by XGBoost, with a measurement of 0.0153 ± 0.0010 s, and Random Forest follows with a measurement of 0.1509 ± 0.0084 s. SVM requires a longer prediction time under the current workstation-based evaluation, indicating a higher computational burden than RF and XGB.

To evaluate the contribution of meta-correlation, an ablation analysis was performed, as shown in Table 6. The results showed comparable performance between the two variants. With the full descriptor, RF achieved 99.06% ± 0.12 accuracy, XGB achieved 98.73% ± 0.17, and SVM achieved 97.78% ± 0.13. Without meta-correlation, RF achieved 98.96% ± 0.19, XGB achieved 98.67% ± 0.19, and SVM achieved 97.71% ± 0.17. These results indicate that the meta-correlation feature provides an auxiliary interaction descriptor rather than a dominant performance driver. Therefore, its role is interpreted as part of the full engineered descriptor representation rather than as an independent physiological marker.

**Table 6:** Ablation comparison of full descriptor and descriptor without meta-correlation.

Model	Full 106-dimensional descriptor Accuracy (%)	Without Meta-Corr Accuracy (%)
XGB	98.73 ± 0.17	98.67 ± 0.19
SVM	97.78 ± 0.13	97.71 ± 0.17
RF	**99.06 ± 0.12**	98.96 ± 0.19

**Fig. 5:**
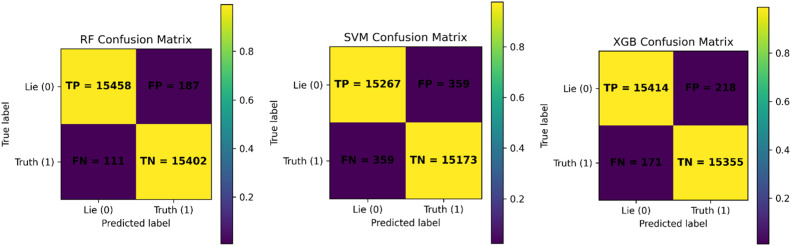
Confusion Matrices of RF, SVM, and XGBoost.

Figure 5 displays confusion matrices that compare the Support Vector Machine (SVM), Random Forest (RF), and XGBoost (XGB) performance. The Random Forest confusion matrix shows equal true positives (15458) and true negatives (15402), with small numbers of false positives (187) and false negatives (111). The system achieved balanced detection performance, with low false-positive and false-negative rates across both Lie and Truth classes. The system showed minimal class prediction errors, misclassifying instances at a rate of less than 2%. XGB shows moderate misclassification, producing 218 false positives and 171 false negatives. XGB shows higher misclassification counts than RF because it produces more false positives and false negatives. The distribution maintains its symmetrical form, which demonstrates that both classes are consistently distinguished from one another. The SVM algorithm produces more errors than ensemble-based methods, yielding 359 false positives and 359 false negatives. The system shows symmetric decision boundary behavior because both FP and FN values are equal, yet the system shows lower separability under the RBF kernel configuration because FP and FN values have different magnitudes.

A structured descriptor space analysis was performed, including Linear Discriminant Analysis (LDA) projection testing to determine whether class discrimination is an essential part of the structured descriptor space. The LDA system establishes a linear transformation that maximizes between-class variability and uses its first discriminant axis (LD1) to minimize within-class variability.

**Fig. 6:**
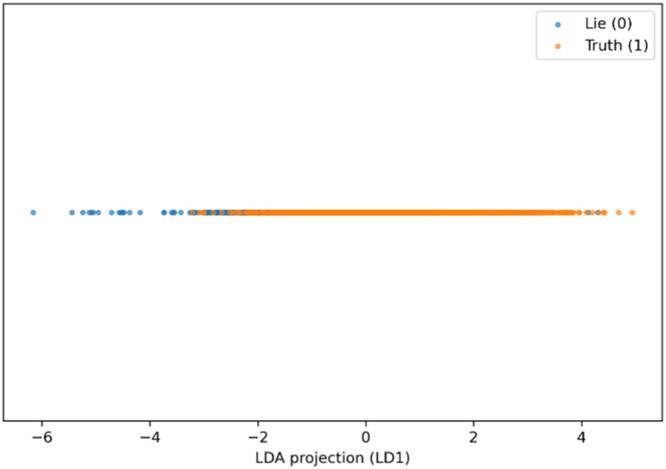
Linear Discriminant Analysis (LDA) Visualization.

Figure 6 demonstrates that the Lie (0) samples and Truth (1) samples create different distribution patterns at the LD1 measurement point. The Truth windows mostly occupy the positive LD1 area, while the Lie windows concentrate their presence in the negative LD1 space. The process shows continuous development, leading to discrimination because feature statistics change gradually rather than showing sudden shifts at cluster boundaries. The central decision area shows some overlap with a range of LD1 values from -3 to -2, but most samples maintain their distribution across different intervals.

**Fig. 7:**
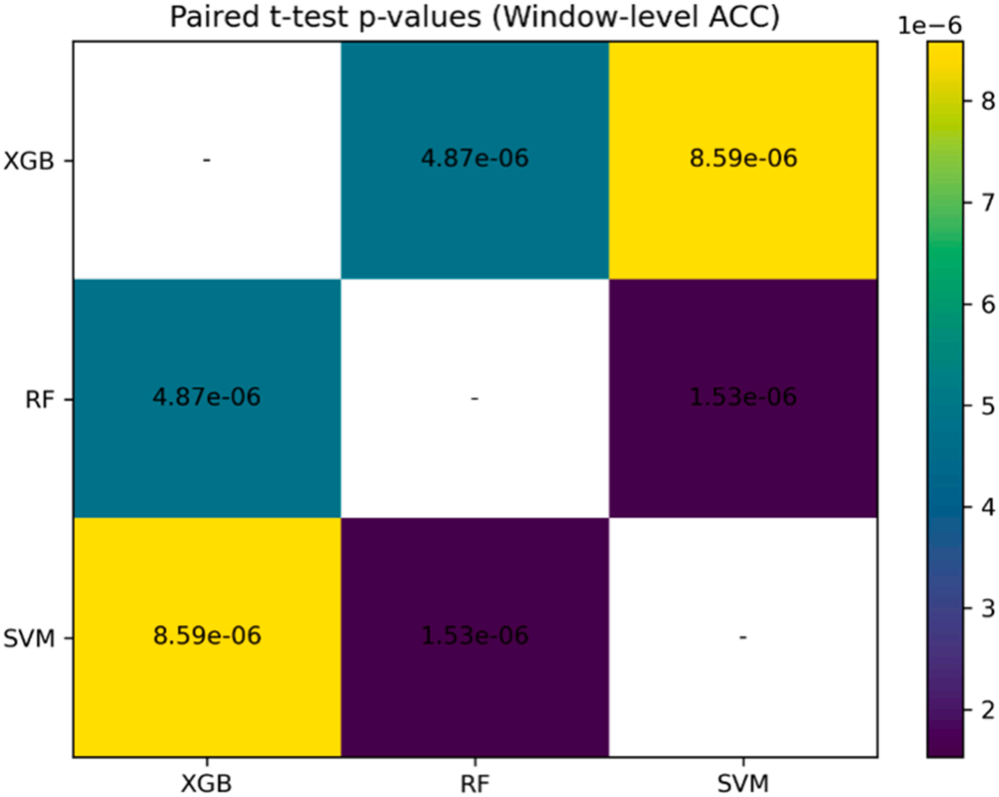
Paired t-test p-value Matrix Based on Fold-Wise Accuracy.

Paired t-tests assessed the statistical significance of performance differences between classifiers, using fold-wise accuracy scores from their stratified 5-fold cross-validation. The results show that all classifier pairs in Figure 7 have p-values below the 0.01 threshold, indicating statistically consistent differences across the five fold-wise accuracy values under the current evaluation setting. The test results show p-values of 4.87 × 10⁻⁶ for XGB vs. RF, 8.59 × 10⁻⁶ for XGB vs. SVM, and 1.53 × 10⁻⁶ for RF vs. SVM. The RF-SVM test results show the smallest p-value, indicating that the two models have the greatest accuracy difference during fold-wise testing. The other tests demonstrated constant low p-values, which suggest that the observed performance differences were consistent across folds under the applied validation protocol.

### Binary model efficiency analysis

The descriptor demonstrates strong window-level accuracy across three learning methods: boosting with XGBoost, bagging with Random Forest, and kernel-based optimization with SVM. Random Forest achieved 99.06% ± 0.12 accuracy, followed by XGBoost (98.73% ± 0.17) and SVM (97.78% ± 0.13). The narrow standard deviations across stratified folds indicate stable convergence across repeated data partitions. The test results are consistent, showing that the discriminative structure exists in the handcrafted descriptor space rather than being tied to a specific classifier architecture.

The arrangement of classifiers in a system directly determines its computational performance. The kernel-based support vector machine requires 264.43 seconds for training and 4.06 seconds for each window during inference when using a 106-dimensional descriptor space. Ensemble models achieve fast model training and short prediction times, while XGB and RF show different time requirements for their operations. The results demonstrate that organizations must choose between two competing needs: they want to achieve accurate predictions, but their advanced systems require high computational resources.

The error distribution analysis provides additional evidence that supports this finding. The confusion matrices show that all models tested display equal performance between the Lie (0) and Truth (1) classification tasks. The ensemble-based methods achieved better performance than SVM because they produced fewer false positives and false negatives through their enhanced capacity to handle feature interactions. The descriptor construction shows no systematic bias towards any class, which proves that class imbalance does not occur in this system.

The complete analysis of the feature space using Linear Discriminant Analysis (LDA) shows that class separation can be partially described by linear functions [32]. The first discriminant axis projection reveals distinct distribution patterns for both Lie and Truth windows, with minimal overlap near the decision boundary. The representation level maintains inter-class variance present before the nonlinear optimization process, thereby supporting the structural connection between different multi-domain descriptor integration methods.

The paired t-tests used for statistical validation show that classifier performance differences are repeatable, as evidenced by their testing results, which demonstrate that all model pairs maintained consistent results throughout evaluation (p < 0.01). The evaluation pipeline demonstrates its methodological evaluation strength through statistical consistency while showing how different learning mechanisms interact with descriptor structure.

**Fig. 8:**
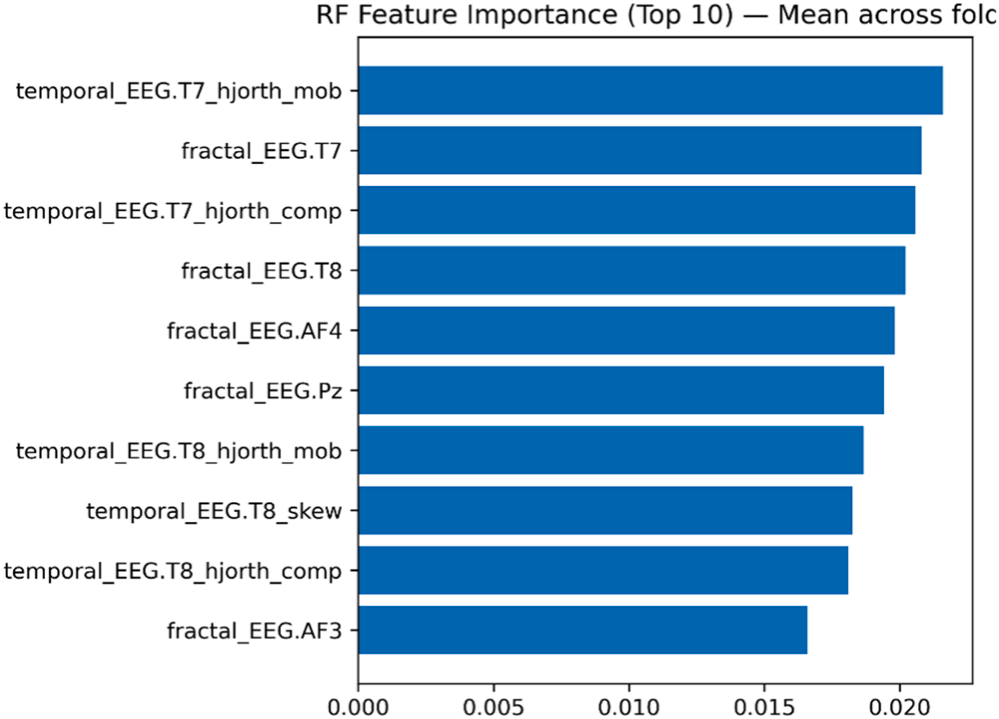
Top-ranked Random Forest feature importance scores for the proposed descriptor.

The value of feature contribution analysis goes beyond understanding descriptor behavior. The Random Forest importance ranking indicates that the most influential descriptors were mainly temporal and fractal features from T7-related channels. The highest-ranked features were mainly temporal descriptors, including Hjorth Activity and standard deviation from T7-related channels. T7-related fractal features achieved high rankings, indicating their contribution to model-level discrimination between Lie and Truth windows. This result should be interpreted cautiously because temporal electrodes may capture both neural and non-neural components, according to the results shown in Fig. 8

**Fig. 9:**
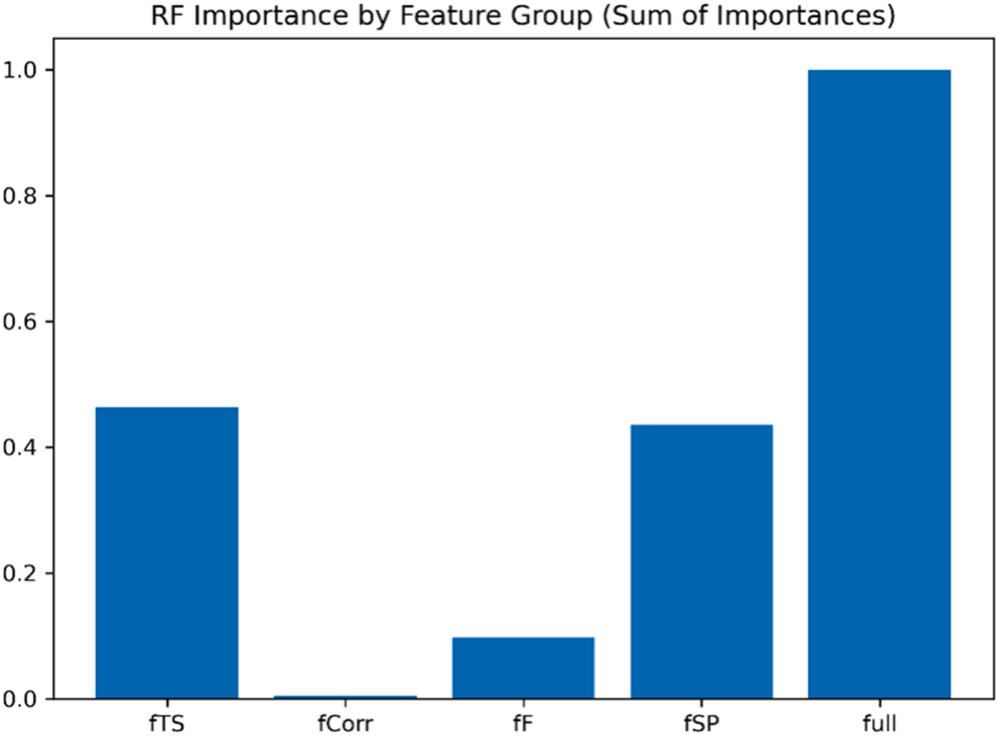
Aggregated Random Forest feature importance by descriptor group.

The analysis of group-level aggregation shows that single-channel temporal and fractal descriptors accounted for 45% of the total aggregated importance, while spectral features account for 31%, and connectivity-based descriptors provide 23% of total importance, according to the results presented in Fig. 9. The distribution pattern indicates that temporal, fractal, spectral, and connectivity-related descriptors contribute differently to the discrimination of experimentally labeled Lie and Truth windows. These feature-importance results should be interpreted as model-level discriminative contributions rather than evidence of condition-specific neurophysiological mechanisms.

To provide contextual validation of the proposed system, this study compared it with previous Lie and Truth detection research that used the same LieWaves dataset for their assessment, as shown in Table 7. Existing approaches include deep learning, hybrid frameworks combining CNN and LSTM methods, EEGNet, fuzzy optimization techniques, and descriptor-based methods using spectral, entropy, and ERP features.

**Table 7:** Comparative Overview of Lie and Truth Detection Approaches on the LieWaves Dataset.

Dataset	Segmentation	preprocessing	Feature	Classifier	Acc (%)
MI-ENFS Neuro-Fuzzy [31]	Full signal	0.5–45 Hz + ICA	DWT + FFT + Type-2 Fuzzy+ Metaheuristic	EEGNet+ InceptionTime-Light + ANFIS	93.8%
LieWaves Original [10]	OSW 384 window, step 32	0.5–45 Hz	DWT+FFT + Statistical	CNN, LSTM	99.88%
Multi-Scale Entropy [2]	Trial-based	DWT decomposition	FE + TSMFE + HMFE	LDA, SVM, kNN, NB	82.74%
Five-Channel Wearable EEG Deception Detection [4]	Stimulus-based	Bandpass++ segmentation	Spectral + ERP	ML/DL hybrid	92%
Proposed	OSW (384, step 16)	0.5–45 Hz bandpass	Structured Multi-Domain: Temporal + Fractal + Spectral + Phase	Random Forest	99.06 ± 0.12

Although the LieWaves Original study reported a slightly higher accuracy (99.88%) [10], the evaluation protocols differ in several important methodological aspects. The proposed framework uses a stratified 5-fold cross-validation method that requires feature normalization within the training folds while maintaining fold-wise separation for model training and feature scaling. The proposed framework prevents normalization leakage by estimating scaling parameters only from the training folds. However, because the evaluation uses highly overlapping window-level samples, the reported performance is interpreted as descriptor separability under the current controlled protocol.

Previous studies present single accuracy measurements that lack details on their accuracy distribution across different testing intervals. The study reports performance results as mean values with standard deviation, showing 99.06% performance with a standard deviation of 0.12. The descriptor representation maintains stable window-level performance under the current controlled evaluation setting, as indicated by the low standard deviation across folds.

The proposed framework delivers competitive performance through its lightweight preprocessing system and the use of understandable handcrafted descriptors.

Table 8 presents an auxiliary portability analysis of the proposed descriptor across heterogeneous EEG binary classification datasets. The additional datasets, including MAT, SAM40, and DASPS, represent different cognitive or affective tasks. Instead, it examines whether the proposed structured descriptor construction can be technically applied across different EEG binary classification settings under consistent preprocessing, feature extraction, and classifier configurations.

**Table 8:** Auxiliary Descriptor Portability Analysis Across Heterogeneous EEG Binary Classification Tasks.

Dataset	Class	Model	Accuracy (%)	Recall (%)	F1 Score (%)	AUC (%)	Training Time (s)	Inference Time (s)
MAT [33]	2	XGB	96.06 ± 0.59	92.85 ± 1.34	94.56 ± 0.88	99.27 ± 0.14	0.948 ± 0.103	0.0018 ± 0.0001
		RF	93.67 ± 0.44	87.68 ± 0.88	90.86 ± 0.71	98.71 ± 0.27	1.021 ± 0.038	0.0876 ± 0.0008
		SVM	98.94 ± 0.20	98.21 ± 0.43	98.58 ± 0.27	99.84 ± 0.12	1.452 ± 0.029	0.1570 ± 0.0041
SAM40 [34]	2	XGB	93.17 ± 1.46	91.28 ± 1.55	92.32 ± 1.63	98.28 ± 0.73	0.7087 ± 0.0378	0.0016 ± 0.0001
		RF	91.94 ± 1.23	88.80 ± 1.67	90.67 ± 1.50	98.59 ± 0.65	0.3849 ± 0.0065	0.0756 ± 0.0047
		SVM	98.78 ± 1.11	98.63 ± 1.16	98.66 ± 1.21	99.66 ± 0.39	0.1891 ± 0.0061	0.0176 ± 0.0007
DASPS [35]	2	XGB	99.56 ± 0.06	99.79 ± 0.02	99.57 ± 0.06	99.99 ± 0.00	38.9810 ± 43.5967	0.0166 ± 0.0013
		RF	99.36 ± 0.11	99.98 ± 0.02	99.36 ± 0.11	99.99 ± 0.00	26.4506 ± 0.7877	0.0366 ± 0.0002
		SVM	96.34 ± 0.33	95.85 ± 0.36	96.32 ± 0.33	99.42 ± 0.09	14.1515 ± 0.3898	0.8419 ± 0.0063
LieWaves [10]	2	XGB	98.73 ± 0.17	98.56 ± 0.21	98.72 ± 0.17	99.90 ± 0.03	6.4046 ± 0.2109	0.0153 ± 0.0010
		SVM	97.78 ± 0.13	97.73 ± 0.15	97.78 ± 0.13	99.66 ± 0.06	264.4311 ± 20.6280	4.0603 ± 0.0735
		RF	99.06 ± 0.12	98.83 ± 0.23	99.06 ± 0.12	99.96 ± 0.01	7.4777 ± 0.0226	0.1509 ± 0.0084

The proposed descriptor achieved high classification accuracy across these heterogeneous binary EEG tasks under the applied evaluation settings. However, these results should be interpreted only as descriptor portability evidence, not as external validation of Lie and Truth EEG patterns. All classifiers-maintained accuracy rates above 91% on both the MAT and SAM40 tests, whereas the DASPS dataset achieved exceptional results, with accuracy exceeding 99%. These results indicate that the descriptor can be technically applied to different EEG binary classification datasets under the applied preprocessing and evaluation settings, as shown by these results.

Despite differences in dataset size and computational demand, ensemble-based models showed low inference time under the workstation-based evaluation setting.

Overall, the results suggest that the proposed descriptor can be applied across several heterogeneous EEG binary classification tasks. These results should be interpreted as evidence of descriptor portability.

## Limitations

The structured multi-domain EEG descriptor framework supports reproducibility through a deterministic operational pipeline. However, several methodological limitations should be acknowledged. First, the validation procedure uses window-based testing with stratified 5-fold cross-validation. This design enables controlled window-level evaluation and supports descriptor-separability analysis in a subject-dependent setting. However, it does not represent strict subject-independent validation and does not evaluate how the model performs on entirely unseen participants. To address this limitation, future work should test inter-subject variability using subject-wise group validation, leave-one-subject-out validation, and cross-session validation.

Second, the evaluation uses the LieWaves dataset, collected with five-channel wearable equipment (AF3, T7, Pz, T8, AF4), to test its performance. This configuration aligns with the wearable EEG scope of the study but limits spatial resolution and whole-brain connectivity modeling. Therefore, phase-based connectivity descriptors are interpreted as engineered pairwise synchronization features rather than comprehensive brain network measures. Future work should evaluate the framework using higher-density EEG recordings.

Third, the handcrafted descriptor combines multiple domains to produce a 106-dimensional feature vector. Although this dimensionality is moderate compared with raw EEG or deep neural feature representations, it may still increase computational demand and overfitting risk in limited-subject EEG datasets. The full descriptor was retained in this study to preserve reproducibility and domain-level interpretability. Future work should investigate structured dimensionality reduction methods.

Fourth, the physiological interpretation of phase-based connectivity and meta-correlation descriptors is limited by the five-channel wearable montage, short-window estimation, and the absence of explicit artifact correction. Therefore, these descriptors are presented as engineered discriminative features rather than validated neurophysiological markers. Future studies using higher-density EEG, artifact-aware preprocessing, and dynamic connectivity analysis are needed to more rigorously evaluate their physiological meaning.

Fifth, the evaluation uses classical machine learning classifiers to provide controlled and interpretable descriptor-level assessment. The 106-dimensional handcrafted descriptor may increase the risk of overfitting when used directly in deep learning models, especially given the limited size of the LieWaves dataset. Although the manuscript includes Random Forest feature importance, descriptor-group analysis, LDA visualization, paired t-test analysis, and ablation evaluation, SHAP-based explanations were not implemented in the current version. Future work should explore lightweight hybrid neural models after dimensionality reduction and incorporate SHAP-based global and local explanations for descriptor contribution analysis.

Sixth, the present study evaluates computational efficiency using workstation-based training and inference time. Hardware-level deployment, RAM profiling, and energy-consumption measurements on wearable edge devices were not conducted in this study and remain necessary for practical implementation. Although the 106-dimensional descriptor is compact, wearable deployment also depends on the computational cost of filtering, Welch PSD estimation, wPLI computation, and repeated feature extraction. These aspects should be examined in future hardware-oriented studies.

Seventh, the current framework uses static connectivity descriptors computed within fixed-length EEG windows. Although this design supports reproducibility and deterministic feature extraction, it does not explicitly model EEG non-stationarity or time-varying connectivity across successive windows. Future work should investigate dynamic connectivity to better capture transient EEG interactions.

The proposed pipeline maintains reproducibility despite existing limitations. It shows the potential research paths to expand EEG acquisition scenarios through developing a descriptor framework.

## Related research article

None.

## For a published article

None.

## Ethics statements

This study conducted a secondary analysis of the publicly accessible LieWaves dataset, which includes anonymized EEG recordings collected by the original study following their institutional ethics approval and informed-consent procedures. No new human participants were recruited, and no additional personal data were collected in this work. The dataset was used in accordance with its published data-sharing and redistribution terms.

## Declaration of generative AI and AI assisted technologies in the writing process

We declare that generative AI tools were used in the preparation of this manuscript. Specifically, Gemini and ChatGPT were employed to assist in organizing and structuring the manuscript, while QuillBot and Grammarly were used to enhance clarity, coherence, and grammar. Following the use of these AI-assisted tools, the authors reviewed, edited, and verified all content independently, and they take full responsibility for the accuracy, integrity, and originality of the work presented in this publication.

## CRediT authorship contribution statement

**Dwi Utari Surya:** Conceptualization, Data curation, Software, Writing – original draft, Writing – review & editing. **Sholeh Hadi Pramono:** Conceptualization, Methodology, Supervision, Validation. **Panca Mudjirahardjo:** Methodology, Project administration, Resources. **Muhammad Aziz Muslim:** Formal analysis, Investigation, Validation. **Cries Avian:** Data curation, Software, Visualization, Writing – review & editing. **Mahdin Rohmatillah:** Data curation, Software, Writing – review & editing.

## Declaration of competing interest

The authors declare that they have no known competing financial interests or personal relationships that could have appeared to influence the work reported in this paper.

## Data Availability

LieWaves data are available at Mendeley Data (https://doi.org/10.17632/5gzxb2bzs2.2). Code: https://github.com/dutarisurya/lie-truth-eeg-descriptor.
